# The Harvey Lectures

**Published:** 1907-03

**Authors:** 


### BOOKS AND AUTHORS.

**The Harvey Lectures.** Delivered under the auspices of the Harvey Society of New York. 1905-1906. Philadelphia and London: J. B. Lippincott Co.

The Harvey Society of New York was organised in the spring of 1905 through the efforts of Dr. Graham Lusk for the purpose of spreading knowledge of the medical sciences through the means of public lectures in annual series dealing with the purely experimental study of medicine, the lectures to be given by those who devote their time to experimental work largely, The lectures are not, as might be supposed, confined to a bare recital of the results achieved by the lecturers themselves, except in rare instances; they are rather a broad presentation of the subject of experimental medicine from the laboratory point of view. The lectures are given under the patronage of the New York Academy of Medicine and present a critical review of the work done in each subject considered in the light of the most recent advances.

The titles of the lectures published in this volume are as follows: Theory of narcosis, Prof. Hans Meyer, University of Vienna; Modern problems of metabolism, Carl van Noorden, University of Vienna; On trypanosomes, Frederick G. Novy, University of Michigan; Autolysis, P. A. Leven, Rockefeller Institute for Medical Research; A critical study of serum therapy, W. H. Park, University and Bellevue Hospital Medical College; The neuroses, Lewellys F. Barker, John Hopkins University; Fatigue, Frederick S. Lee, Columbia University; The formation of uric acid, Lafayette B. Mendel, Yale; The extent and limitations of the power to regenerate in man and other vertebrates, T. H. Morgan, Columbia.

